# Implementing Vertical Federated Learning Using Autoencoders: Practical Application, Generalizability, and Utility Study

**DOI:** 10.2196/26598

**Published:** 2021-06-09

**Authors:** Dongchul Cha, MinDong Sung, Yu-Rang Park

**Affiliations:** 1 Department of Biomedical Systems Informatics Yonsei University College of Medicine Seoul Republic of Korea; 2 Department of Otorhinolaryngology Yonsei University College of Medicine Seoul Republic of Korea

**Keywords:** federated learning, vertically incomplete data, privacy, machine learning, coding, data, performance, model, security, training, dataset, unsupervised learning, data sharing, protection

## Abstract

**Background:**

Machine learning (ML) is now widely deployed in our everyday lives. Building robust ML models requires a massive amount of data for training. Traditional ML algorithms require training data centralization, which raises privacy and data governance issues. Federated learning (FL) is an approach to overcome this issue. We focused on applying FL on vertically partitioned data, in which an individual’s record is scattered among different sites.

**Objective:**

The aim of this study was to perform FL on vertically partitioned data to achieve performance comparable to that of centralized models without exposing the raw data.

**Methods:**

We used three different datasets (Adult income, Schwannoma, and eICU datasets) and vertically divided each dataset into different pieces. Following the vertical division of data, overcomplete autoencoder-based model training was performed for each site. Following training, each site’s data were transformed into latent data, which were aggregated for training. A tabular neural network model with categorical embedding was used for training. A centrally based model was used as a baseline model, which was compared to that of FL in terms of accuracy and area under the receiver operating characteristic curve (AUROC).

**Results:**

The autoencoder-based network successfully transformed the original data into latent representations with no domain knowledge applied. These altered data were different from the original data in terms of the feature space and data distributions, indicating appropriate data security. The loss of performance was minimal when using an overcomplete autoencoder; accuracy loss was 1.2%, 8.89%, and 1.23%, and AUROC loss was 1.1%, 0%, and 1.12% in the Adult income, Schwannoma, and eICU dataset, respectively.

**Conclusions:**

We proposed an autoencoder-based ML model for vertically incomplete data. Since our model is based on unsupervised learning, no domain-specific knowledge is required in individual sites. Under the circumstances where direct data sharing is not available, our approach may be a practical solution enabling both data protection and building a robust model.

## Introduction

Machine learning (ML) is widely deployed in our daily lives, including, but not limited to, personalized digital media, product recommendations, and health care services. Building high-quality ML models requires a huge amount of data for training [1]. Conventional ML algorithms typically require the training data to reside where the models are trained. Recently, there has been an increasing level of concern about data privacy [2]. The EU General Data Protection Regulation and the US Health Insurance Portability and Accountability Act are examples of regulations to secure sensitive information when gathering such information centrally. Moreover, as more data are needed for a robust ML model, raw data are a crucial asset. Sharing raw data raises data governance issues, making data owners hesitant about sharing their data.

An alternative approach to overcome such concerns is federated learning (FL). FL is a learning process in which the individual data owners train a model collaboratively without exposing the original data to others [2]. For the protection of data privacy, k-anonymity [3], l-diversity [4], and t-closeness [5] are well-established methods. Differential privacy [6] is another semantic method to add noise to data. Using such methods enables the aggregation of perturbed data with fewer concerns of exposing the original data. However, stronger protection of privacy requires stronger perturbations of the original data, which reduces the utility; in other words, this results in low-quality ML models. An alternative approach is homomorphic encryption [7], which offers training with encrypted data. However, training such a model is relatively slow, possibly making it impractical to be used in real-world applications [8].

**Figure 1:**
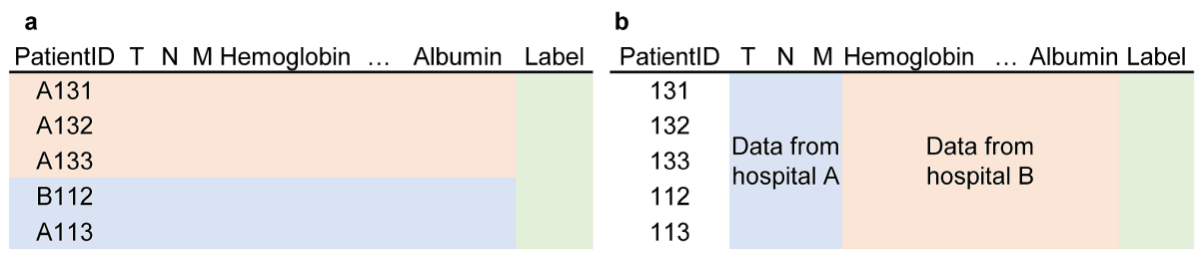
Classification of federated learning. Assume a colorectal cancer patient dataset, and only the target label is gathered centrally. (a) In horizontally partitioned data, patients share the same feature space, but features are collected at different sites. (b) In vertically partitioned data, patient features are present in different sites.

FL could be divided into horizontal and vertical frameworks [2]. In horizontal FL, the data have the same feature space but are distributed among different organizations. In other words, all rows share the same columns but could originate from different sites. In contrast, vertical FL takes vertically partitioned data for training. For each row in the database, columns (features) originate from several different sites. Consider a database of colorectal cancer patients consisting of tumor-node-metastasis staging and laboratory results gathered from different hospitals, and we want to build an ML model to predict survival. In the horizontal FL setting, different organizations train ML models in their individual databases but share the same feature space (Figure 1a). However, in the vertical FL setting, individual tests are spread among different hospitals (eg, tumor stage in hospital A and laboratory tests in hospital B), and ML training is performed without aggregation of raw patient data (Figure 1b). Study results based on horizontal FL [9,10] show comparable performance to that of ML models trained centrally. For vertical FL, there is the possibility of logistic regression [11], linear regression [12], boosting model [13], a model capable of linear and logistic regressions, and neural network models [14].

We here present a simple, practical, robust, and novel vertical FL method based on autoencoder neural networks [15], more specifically, an overcomplete autoencoder, in which hidden layers have a higher dimension than input layers. We tested our method in three datasets, including two medical datasets, to demonstrate generalizability and utility.

## Methods

### Overcomplete Autoencoder for the Latent Representation of Original Data

**Figure 2:**
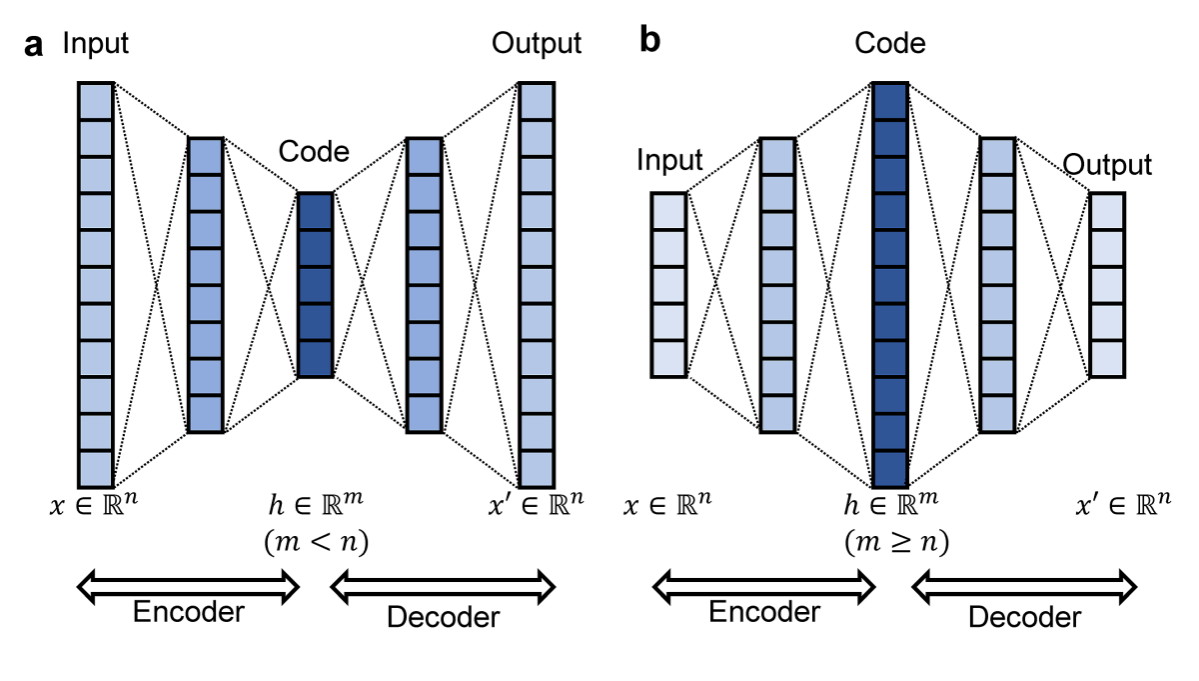
The autoencoder network, which is an unsupervised machine learning algorithm. Input and output are the same; thus, they have identical feature space. (a) The conventional autoencoder has a latent space dimension smaller than the input space (m<n). (b) The overcomplete autoencoder has equal or higher dimensions in the latent space (m≥n).

An autoencoder is a feed-forward neural network with the same inputs and outputs that are trained in an unsupervised manner. The network is fully connected and consists of an encoder and a decoder. The encoder transforms the input into a latent representation, and the decoder maps the latent representation back to the original input. During training, the machine learns both the encoder’s and decoder’s weights by minimizing the reconstruction loss. There are three main layers of an autoencoder: an input layer, hidden (including code) layer, and output layer. By adding a hidden layer with constraints such as fewer dimensions than the given input (Figure 2a, h ∈
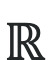
*^m^* [*m*<*n*]) the machine tries to learn essential features in the given input. Since a conventional autoencoder reduces dimension, there is an inevitable loss of information. In an overcomplete autoencoder, hidden layers are larger than or equal to the input layer (Figure 2b, h ∈
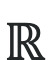
*^m^* [*m*≥*n*]). By having more feature space in the code layer, information loss could be minimized, especially when datasets have a small number of features. Additionally, latent representation differs from the original input data, enabling both security and performance.

### Datasets and Vertical Division of Data

#### Adult Income Dataset

The adult income dataset [16] has two labels: whether or not a person earns over 50,000 per year, with eight categorical and six continuous variables as input variables. The dataset included 37,155 individuals with a salary ≤50,000 and 11,687 individuals with a salary >50,000 per year. We randomly sampled from the 11,687 individuals with a salary under 50,000 to balance the dataset (random undersampling), so that the total dataset comprised 23,374 individuals, and set the prediction chance level to 50%. We vertically divided this dataset into three pieces, assuming three different organizations possessing partial data over individuals (Table 1).

**Table 1:** Dataset composition and training parameters with division to simulate vertically partitioned data.

Dataset	Division	Dataset size (number of rows)	Feature dimension	Autoencoder layers	Aggregated dimension
Adult income	3 sites	23,374	5, 5, 4	64-128-64	384×23,374
Schwannoma	3 sites	50	7, 3, 5	64-128-64	384×50
eICU	7 sites	15,762	3, 4, 9, 3, 3, 4, 6	64-128-64	896×15,762

#### Vestibular Schwannoma Dataset

The vestibular schwannoma dataset [17] is an anonymized, private, medical dataset to predict hearing disabilities following surgery. We included this dataset to demonstrate its feasibility in a relatively low number of training samples with sparse data. The dataset included 50 patients, one categorical variable, 14 continuous variables as input, and binary classification labels as output. Since the dataset had 22 and 28 binary target labels, no additional undersampling was performed. The data were vertically split into three sites (Table 1).

#### The eICU Collaborative Research Database

The eICU collaborative research database [18] is a database containing variables used in deriving Acute Physiologic Assessment and Chronic Health Evaluation (APACHE) [19] scores to predict a given patient’s mortality (binary classification). The initial database contained 148,532 intensive care unit (ICU) stays with APACHE version IVa. We only included ICU stays with more than 15 (62.5%) nonnull values, excluding 712 ICU stays. We also excluded 15,968 rows without labels. Therefore, a total of 131,852 rows (ICU stays) were used, 7881 of which were labeled as expired. We randomly picked 7881 alive rows to rule out the class imbalance problem, making the baseline dataset contain 15,762 rows, and vertically divided the dataset into 7 sites (Table 1).

### Training Workflow and Parameters

**Figure 3:**
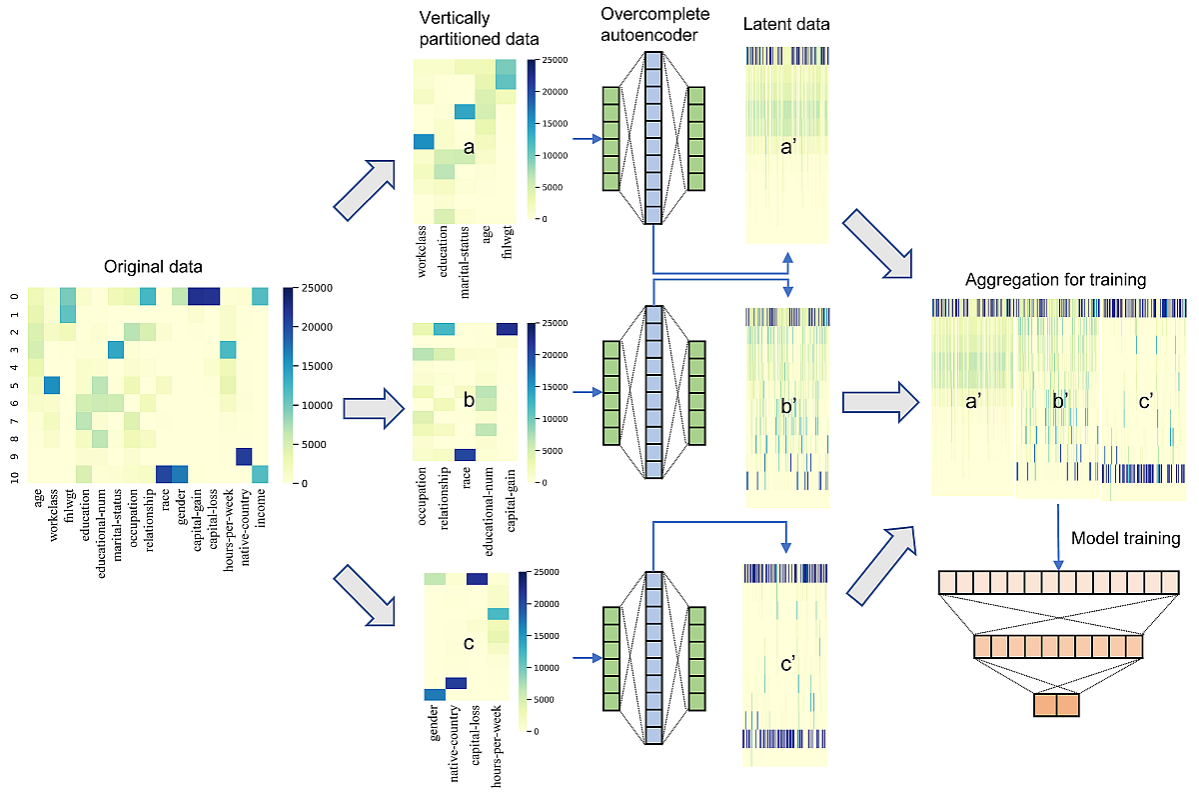
The workflow of vertical federated learning using overcomplete autoencoder. The UCI adult income dataset is illustrated as an example. The dataset consists of 14 features and 1 target label (income). Original data are vertically divided into several datasets, three in this case, to assume data distribution among different sites. The heatmaps show each feature with prevalence. Each site (a, b, c) trains an autoencoder and transmits latent data, which are differently distributed, as seen in the heatmaps (a’, b,’ c’). The latent data are aggregated for training to a server, and the server performs model training. The accuracy of models created using the original data versus aggregated latent data is compared.

All three datasets were vertically divided. In all three datasets, we assumed that a third-party relay server performs data alignment between different servers. For example, the third row in server A is also the third row in servers B and C. To test the generalizability of our approach, we divided the dataset into various numbers (Table 1). Following the vertical division of data, overcomplete autoencoder-based model training was performed for each site (Figure 3 a, b, c). Following training, each site’s latent data (Figure 3 a’, b,’ c,’ representations in the code layer) were aggregated for training. We used PyTorch [20] with the Fastai [21] library for this task. Each site was vertically divided to simulate vertically partitioned data among different sites. Accuracy and area under the receiver operating characteristic curve (AUROC) were used as the evaluation metrics for classification tasks.

Autoencoder models were trained using an initial learning rate of 0.01 and a learning rate decay of 0.99. There is a concern that the ML algorithm might learn an identity function, which may not correctly perturb (or encode) the data. However, a previous study [22] using stochastic gradient descent (SGD) when training resulted in a useful data representation. In addition to using SGD, we also used a weight decay of 0.1 to prevent the autoencoder models from learning the identity function and overfitting.

A tabular neural network model with categorical embedding was used when training. A centrally based model was used as a baseline model. For each vertically split data, both models were trained: an ML model based on each vertically split dataset (Figure 3 a, b, c) and an ML model based on latent representations of each split dataset (Figure 3 a’, b’, c’). Finally, the central-based model was compared to the latent data aggregated model for benchmarking our vertical federated neural network model.

### Code Availability

Since autoencoders are widely implemented in various environments, we do not offer the source code publicly. However, codes will be available upon request to the corresponding author for noncommercial, educational purposes.

## Results

### Transformation of the Data to Latent Representations

The autoencoder-based network successfully transformed the original data into latent representations with no domain knowledge applied. These altered data were different from the original data in terms of both the feature space and data distributions (Figure 3 a’, b’, c’), indicating appropriate data security.

### Classification Performance

Following latent data aggregation, we tested the built model against centralized models and individually trained models using the vertically incomplete original data and latent data (Table 2; see Multimedia Appendix 1 for a detailed division of the feature space). The performance of the autoencoder increased as the number of the code layers increased (see Multimedia Appendix 2 for detailed results). Of note, since we used categorical embeddings when putting categorical variables to the autoencoder and tabular neural network model, latent representations of the original data were continuous variables. The adult income and eICU datasets, which have a relatively large number of rows, did not suffer from a fluctuation of accuracy and AUROC. Although the schwannoma dataset, which only has 50 rows, showed a fluctuation of accuracy and AUROC among different sites, the overall accuracy and AUROC penalties were still acceptable. The eICU dataset had abundant feature space and was vertically divided into seven sites. There was still a minimal loss of accuracy and AUROC, implying good utility while preserving data privacy (Table 2).

**Table 2:** Classification results of the three datasets.

Site		Adult income dataset			Schwannoma dataset			eICU dataset	
		Accuracy	AUROC^a^	Accuracy		AUROC	Accuracy		AUROC
**Central**									
	Before VFL^b^	0.83	0.91	0.90		0.84	0.81		0.89
	After VFL^c^	0.82	0.90	0.82		0.84	0.80		0.88
	Difference^d^	–1.20	–1.10	–8.89		0	–1.23		–1.12
**A**									
	Before VFL	0.81	0.89	0.82		0.81	0.70		0.72
	After VFL	0.77	0.83	0.78		0.86	0.70		0.72
	Difference	–4.94	–6.74	–4.88		+6.17	0		0
**B**									
	Before VFL	0.81	0.90	0.76		0.82	0.73		0.80
	After VFL	0.77	0.83	0.78		0.83	0.72		0.79
	Difference	–4.94	–7.78	+2.63		+1.22	–1.37		–1.25
**C**									
	Before VFL	0.67	0.73	0.48		0.60	0.55		0.57
	After VFL	0.76	0.83	0.62		0.71	0.56		0.57
	Difference	+13.43	+13.70	+29.17		+18.33	1.82		0

^a^AUROC: area under the receiver operating characteristics curve.

^b^VFL: vertical federated learning.

^c^Corresponding to the latent representation of original data (central, A, B, or C) in the code layer.

^d^The difference is compared between AUROCs in classification tasks.

## Discussion

### Principal Results

We have successfully transformed original data into latent representations and trained ML models with perturbed data, resulting in minimal loss of accuracy while preserving data privacy. In an autoencoder network, ML models learn data representation in an unsupervised manner. Therefore, no domain knowledge is required to train the model. Since the code layer has more layers than the input layer, resulting in high dimensionality, this method requires more computing power compared to that required for traditional autoencoders. However, loss of information is minimal, even though the data are severely perturbed (Figure 3 a’, b’, c’). Although slight, there was still a loss of accuracy and AUROC in the trained ML model (Table 2). We suspect this was due to redundant information generated by the network, which acts as noise when training an ML model. The model’s design is somewhat similar to local differential privacy [23] in that each site performs training of ML models independently before sending the perturbed data to a central server. The main difference is that differential privacy has an equal number of feature space dimensions as in the original dataset, whereas our approach alters the feature space to a predefined number of hidden layers.

To check its generalizability, we tested three different datasets with various vertically split datasets. Training the ML model worked well in all datasets, even with a relatively small number of rows. Moreover, some datasets were vigorously divided, but the accuracy remained comparable to that of the centralized ML model. In real-life practice, our model may enable building an ML model without the direct exchange of sensitive information among different data owners. For example, a patient may undergo some routine complete blood count test in one hospital, obtain imaging studies in another, and perform electrolyte tests in the other hospital. When building a classifier model, three sites (hospitals) may train our proposed model individually and share the latent feature space to train the model without directly exposing the patient’s data.

### Comparison With Prior Work and Limitations

Earlier works on federated ML using vertically partitioned data focused on the logistic regression [11], linear regression [12], and boosting [13] models. Hardy et al [12] also utilized additively homomorphic encryption. In their study, both nonprivate and federated settings showed the same accuracy, AUROC, and F1 score. However, the training time was in the order of hours per epoch in high-performance cloud-based machines, which may not be practical. Cheng et al [13] proposed SecureBoost, which exhibited a performance comparable to that of nonprivacy-preserving gradient boosting machine models. They theoretically proved that if both ML models have identical initialization and parameters, the SecureBoost algorithm is lossless; that is, the model shows comparable accuracy to the nonfederated boosting model. Mohassel et al [14] suggested a system capable of linear regression, logistic regression, and neural networks. They used a secure multiparty computation [24] framework with two noncolluding servers (secure two-party computation) to train ML models in a privacy-preserving fashion. The results were promising, but the authors suggested that the neural network model is not yet practical due to the high number of interactions and communications costs.

In this study, we assumed that each client performs autoencoder-based data alteration; therefore, file transmission happens only once when building an ML model. Continuous network connections are not necessary. In addition, training an overcomplete autoencoder is not computationally expensive, which makes our proposed model practical. Similar to other privacy-preserving methods, our model ensures no data leakage beyond data owners. Moreover, we have demonstrated that our approach enables more than two participants to aggregate the latent data, allowing more features per person as the number of participating institutions increases.

Our study has limitations. First, even though the data are differently shaped, data owners still need to transmit the coded data to a central location, which may have room for reverse engineering. However, unless the original feature space is revealed to the recipient, reverse engineering may be difficult. Moreover, the latent space is much bigger than the original feature space, making data transmission redundant. Given sufficient network capacity, this should not be a critical issue. Second, more rigorous results are genuinely needed using cross-validation. Last but not least is the explainability of the model. Since the model transforms feature space into latent space, each feature’s meaning in the aggregated data is somewhat different; it cannot be directly associated with the original feature space. Indirectly, site-wise comparison of accuracy using only part of available data could be used to measure feature importance, but future studies should be performed to overcome this limitation.

### Conclusions

We proposed an overcomplete autoencoder–based ML model for vertically incomplete data. Since our model is based on unsupervised learning, no domain-specific knowledge is required in individual sites. Under the circumstances where direct data sharing is not available, our approach may be a practical solution enabling both data protection and building a robust model.

## Appendix

Multimedia Appendix 1 Feature distribution of individual sites in three datasets.

Multimedia Appendix 2 Classification results of the three datasets, and comparison with conventional undercomplete autoencoders.

## Abbreviations

APACHE: Acute Physiologic Assessment and Chronic Health Evaluation
AUROC: area under the receiver operating characteristics curve
FL: federated learning
ICU: intensive care unit
ML: machine learning
SGD: stochastic gradient descent
