# Supplementary material for: Implementing Vertical Federated Learning Using Autoencoders: Practical Application, Generalizability, and Utility Study
Source: JMIR Med Inform. 2021 Jun 9;9(6):e26598. doi: 10.2196/26598 (PMC8262549; doi:10.2196/26598)
Supplement: Multimedia Appendix 1 [file medinform_v9i6e26598_app1.docx]

| Dataset | Site | Features (Continuous) | Features (Categorical) | Target |
| --- | --- | --- | --- | --- |
| Adult | Central | 'age','fnlwgt','educational-num','capital-gain','capital-loss','hours-per-week' | 'workclass', 'education', 'marital-status', 'occupation', 'relationship', 'race', 'gender', 'native-country' | ‘income’ (>50K, <=50K) |
|  | VFL | #0 to #383 |  |  |
|  | A | ‘age’,’fnlwgt’ | ‘workclass’, ‘education’, ‘marital-status’ |  |
|  | B | ‘educational-num’,’capital-gain’ | ‘occupation’, ‘relationship’, ‘race’ |  |
|  | C | ‘capital-loss’,’hours-per-week’ | ‘gender’, ‘native-country’ |  |
|  | A’ to C’ | #0 to #127 |  |  |
| Schwannoma | Central | 'PTA (250)', ‘PTA (500)', 'PTA (1K)', 'PTA (2K)', 'PTA (3K)', ‘PTA (4K)', 'PTA (8K)', 'SRT', 'WRS', 'MCL', ‘1-5interv','TumSize','VEMP-assym','Caloric-CP' | Approach’ | ‘preserved’ (yes, no) |
|  | VFL | #0 to #383 |  |  |
|  | A | 'PTA (250)', ‘PTA (500)', 'PTA (1K)', 'PTA (2K)', 'PTA (3K)', ‘PTA (4K)', 'PTA (8K)' |  |  |
|  | B | 'SRT', 'WRS', 'MCL' |  |  |
|  | C | ‘1-5interv','TumSize','VEMP-assym','Caloric-CP' | ‘Approach' |  |
|  | A’ to C’ | #0 to #127 |  |  |
| eICU | Central | 'urine','wbc','temperature','respiratoryrate','sodium','heartrate','meanbp','ph','hematocrit','creatinine','albumin','pao2','pco2','bun','glucose', 'bilirubin', 'fio2' | 'intubated', 'vent', 'dialysis', 'eyes', 'motor','verbal','meds',’aids','hepaticfailure','lymphoma','metastaticcancer','leukemia','immunosuppression', 'cirrhosis','diabetes' | ‘actualicumortality’ (yes, no) |
|  | VFL | #0 to #895 |  |  |
|  | A |  | 'intubated', 'vent', 'dialysis' |  |
|  | B | 'respiratoryrate','sodium','heartrate','meanbp','ph' |  |  |
|  | C |  | 'meds',’aids','hepaticfailure','lymphoma','metastaticcancer','leukemia','immunosuppression', 'cirrhosis','diabetes' |  |
|  | D |  | 'eyes', 'motor','verbal' |  |
|  | E | 'urine','wbc','temperature' |  |  |
|  | F | 'hematocrit','creatinine','albumin','pao2' |  |  |
|  | G | 'pco2','bun','glucose', 'bilirubin', 'fio2' |  |  |
|  | A’ to G’ | #0 to #127 |  |  |
