# Supplementary material for: Implementing Vertical Federated Learning Using Autoencoders: Practical Application, Generalizability, and Utility Study
Source: JMIR Med Inform. 2021 Jun 9;9(6):e26598. doi: 10.2196/26598 (PMC8262549; doi:10.2196/26598)
Supplement: Multimedia Appendix 2 [file medinform_v9i6e26598_app2.docx]

The difference is compared between AUROCs in classification tasks. A’ corresponds to the latent representation of data A in the code layer. VFL: Vertical federated learning(with 128 code layers), AUROC: area under the receiver operating characteristics curve. VFL-8: VFL with 8 code layers, VFL-16: VFL with 16 code layers, VFL-32: VFL with 32 code layers. All autoencoder layers are designed as [64–code–64] fashion. Therefore, VFL with 64 code layers are not considered.

| Dataset | Site | Accuracy | AUROC | Site | Accuracy | AUROC | Difference(%) | | |
| --- | --- | --- | --- | --- | --- | --- | --- | --- | --- |
|  |  |  |  |  |  |  | Accuracy | AUROC |  |
| **Adult** | **Central** | **0.83** | **0.91** | **VFL** | **0.82** | **0.90** | **-1.20** | **-1.10** |  |
|  | A | 0.81 | 0.89 | A’ | 0.77 | 0.83 | -4.94 | -6.74 |  |
|  | B | 0.81 | 0.90 | B’ | 0.77 | 0.83 | -4.94 | -7.78 |  |
|  | C | 0.67 | 0.73 | C’ | 0.76 | 0.83 | +13.43 | +13.70 |  |
|  |  |  |  | **VFL-8** | **0.75** | **0.84** | **-9.64** | **-7.69** |  |
|  |  |  |  | A’ | 0.67 | 0.72 | -17.28 | -19.10 |  |
|  |  |  |  | B’ | 0.67 | 0.72 | -17.28 | -20.00 |  |
|  |  |  |  | C’ | 0.67 | 0.72 | 0.00 | -1.37 |  |
|  |  |  |  | **VFL-16** | **0.79** | **0.87** | **-4.82** | **-4.40** |  |
|  |  |  |  | A’ | 0.67 | 0.73 | -17.28 | -17.98 |  |
|  |  |  |  | B’ | 0.69 | 0.75 | -14.81 | -16.67 |  |
|  |  |  |  | C’ | 0.67 | 0.73 | 0.00 | 0.00 |  |
|  |  |  |  | **VFL-32** | **0.78** | **0.87** | **-6.02** | **-4.40** |  |
|  |  |  |  | A’ | 0.68 | 0.74 | -16.05 | -16.85 |  |
|  |  |  |  | B’ | 0.67 | 0.73 | -17.28 | -18.89 |  |
|  |  |  |  | C’ | 0.67 | 0.71 | 0.00 | -2.74 |  |
| **Schwannoma** | **Central** | **0.90** | **0.84** | **VFL** | **0.82** | **0.84** | **-8.89** | **0** |  |
|  | A | 0.82 | 0.81 | A’ | 0.78 | 0.86 | -4.88 | +6.17 |  |
|  | B | 0.76 | 0.82 | B’ | 0.78 | 0.83 | +2.63 | +1.22 |  |
|  | C | 0.48 | 0.60 | C’ | 0.62 | 0.71 | +29.17 | +18.33 |  |
|  |  |  |  | **VFL-8** | **0.64** | **0.79** | **-28.89** | **-5.90** |  |
|  |  |  |  | A’ | 0.80 | 0.79 | -2.44 | -1.97 |  |
|  |  |  |  | B’ | 0.76 | 0.85 | 0.00 | +3.08 |  |
|  |  |  |  | C’ | 0.7 | 0.66 | +45.83 | +10.33 |  |
|  |  |  |  | **VFL-16** | **0.72** | **0.85** | **-20.00** | **+1.07** |  |
|  |  |  |  | A’ | 0.7 | 0.81 | -14.63 | -0.56 |  |
|  |  |  |  | B’ | 0.68 | 0.81 | -10.53 | -1.53 |  |
|  |  |  |  | C’ | 0.7 | 0.74 | +45.83 | +23.03 |  |
|  |  |  |  | **VFL-32** | **0.8** | **0.85** | **-11.11** | **+1.62** |  |
|  |  |  |  | A’ | 0.82 | 0.80 | 0.00 | -0.65 |  |
|  |  |  |  | B’ | 0.82 | 0.80 | +7.89 | -2.58 |  |
|  |  |  |  | C’ | 0.6 | 0.70 | +25.00 | +16.48 |  |
|  |  |  |  |  |  |  |  |  |  |
| **eICU** | **Central** | **0.81** | **0.89** | **VFL** | **0.80** | **0.88** | **-1.23** | **-1.12** |  |
|  | A | 0.70 | 0.72 | A’ | 0.70 | 0.72 | 0 | 0 |  |
|  | B | 0.73 | 0.80 | B’ | 0.72 | 0.79 | -1.37 | -1.25 |  |
|  | C | 0.55 | 0.57 | C’ | 0.56 | 0.57 | 1.82 | 0 |  |
|  | D | 0.71 | 0.76 | D’ | 0.70 | 0.76 | -1.41 | 0 |  |
|  | E | 0.68 | 0.75 | E’ | 0.68 | 0.74 | 0 | -1.33 |  |
|  | F | 0.73 | 0.80 | F’ | 0.72 | 0.78 | -1.37 | -2.50 |  |
|  | G | 0.72 | 0.79 | G’ | 0.71 | 0.78 | -1.39 | -1.27 |  |
|  |  |  |  | **VFL-8** | **0.79** | **0.87** | **-2.47** | **-2.25** |  |
|  |  |  |  | A’ | 0.71 | 0.73 | +1.43 | +1.39 |  |
|  |  |  |  | B’ | 0.69 | 0.77 | -5.48 | -3.75 |  |
|  |  |  |  | C’ | 0.54 | 0.57 | -1.82 | 0.00 |  |
|  |  |  |  | D’ | 0.69 | 0.75 | -2.82 | -1.32 |  |
|  |  |  |  | E’ | 0.65 | 0.71 | -4.41 | -5.33 |  |
|  |  |  |  | F’ | 0.69 | 0.75 | -5.48 | -6.25 |  |
|  |  |  |  | G’ | 0.70 | 0.77 | -2.78 | -2.53 |  |
|  |  |  |  | **VFL-16** | **0.78** | **0.87** | **-3.70** | **-2.25** |  |
|  |  |  |  | A’ | 0.70 | 0.72 | 0.00 | 0.00 |  |
|  |  |  |  | B’ | 0.71 | 0.78 | -2.74 | -2.50 |  |
|  |  |  |  | C’ | 0.54 | 0.55 | -1.82 | -3.51 |  |
|  |  |  |  | D’ | 0.70 | 0.75 | -1.41 | -1.32 |  |
|  |  |  |  | E’ | 0.66 | 0.73 | -2.94 | -2.67 |  |
|  |  |  |  | F’ | 0.69 | 0.76 | -5.48 | -5.00 |  |
|  |  |  |  | G’ | 0.69 | 0.76 | -4.17 | -3.80 |  |
|  |  |  |  | **VFL-32** | **0.79** | **0.87** | **-2.47** | **-2.25** |  |
|  |  |  |  | A’ | 0.70 | 0.72 | 0.00 | 0.00 |  |
|  |  |  |  | B’ | 0.70 | 0.77 | -4.11 | -3.75 |  |
|  |  |  |  | C’ | 0.53 | 0.55 | -3.64 | -3.51 |  |
|  |  |  |  | D’ | 0.69 | 0.74 | -2.82 | -2.63 |  |
|  |  |  |  | E’ | 0.67 | 0.72 | -1.47 | -4.00 |  |
|  |  |  |  | F’ | 0.70 | 0.76 | -4.11 | -5.00 |  |
|  |  |  |  | G’ | 0.72 | 0.79 | 0.00 | 0.00 |  |
